# Imaging before cardiac resynchronisation therapy implantation—luxury or necessity?

**DOI:** 10.1007/s12471-018-1140-2

**Published:** 2018-08-07

**Authors:** A. H. Maass, S. C. Yap

**Affiliations:** 1 Department of Cardiology, Thoraxcenter, University Medical Center Groningen, University of Groningen, Groningen, The Netherlands; 2 Department of Cardiology, Thoraxcenter, Erasmus Medical Center, Rotterdam, The Netherlands

Cardiac resynchronisation therapy (CRT) is increasingly being used to treat or prevent heart failure in patients with intraventricular or atrioventricular conduction delay. Due to improved implantation tools and improved electrode design, implantation failure rates have decreased in recent years from 5–10% to less than 4%, questioning the need for pre-implantation imaging [1]. The introduction of quadripolar leads in particular has led to reduction of implant failures and the need for reoperation due to left ventricular lead failure, as has been shown by randomised comparison of bipolar to quadripolar leads [1].

The success of CRT, however, not only depends on successful implantation but also on several other factors including left ventricular lead position. To predict the optimal implantation site for left ventricular electrodes and to assess availability of target veins in this optimal region, cardiac imaging can be particularly useful.

In this issue of the Netherlands Heart Journal, Nguyên et al. describe their protocol employing computed tomography angiography (CTA) to visualise coronary sinus anatomy prior to CRT implantation [2]. On the one hand, it is important to use a special protocol for the visualisation of coronary veins in patients with heart failure, as it takes longer for contrast medium to travel to the coronary venous system. On the other hand, many patients with heart failure also have impaired renal function, which limits the use of large amounts of contrast medium. With the protocol described by Nguyên et al., there was a high concordance (85%) between CTA and fluoroscopic coronary venogram during implantation. Several veins were only visible during CTA (10%), whereas others were only visible at implant fluoroscopy (5%), demonstrating the additive value of both techniques.

There are several techniques to visualise coronary venous anatomy before implantation. Fluoroscopic imaging of the coronary sinus can be done as a stand-alone procedure but due to the invasive nature of this technique it is usually deferred to the implantation procedure. The caveat of intra-procedural imaging, however, is that an unfavourable anatomy is first discovered during implantation and additional technical resources to overcome the difficulties may not be available. A relatively easy technique to visualise coronary venous anatomy is the venous phase of coronary angiograms. In the work-up of patients with heart failure, coronary angiograms are often performed to rule out underlying coronary artery disease and interventional cardiologists should be aware that the venous phase visualises coronary veins without the need of extra contrast medium and with little increase in radiation exposure of the patient. It has been shown that the information gained from previous coronary angiograms is comparable with retrograde fluoroscopy and in some cases implantation can be performed without additional intra-procedural imaging [3]. CTA as described by Nguyên et al. is a non-invasive technique that not only supports the visualisation of infarcted areas but also demonstrates the anatomy of the phrenic nerve in relation to possible target veins [4]. Despite the incremental radiation exposure, CTA should be considered in all patients, especially in patients with prior myocardial infarction and in patients who did not have a venous phase coronary angiogram.

**Fig. 1:**
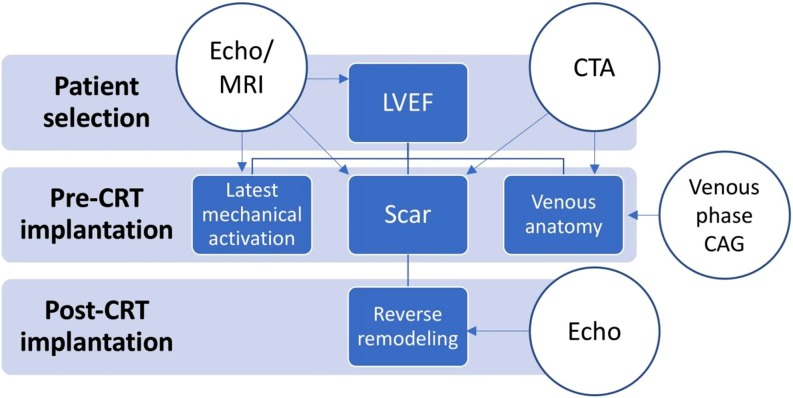
Flow diagram of imaging options before and after CRT implantation. *CTA* computed tomography angiography; *MRI* magnetic resonance imaging; *LVEF* left ventricular ejection fraction; *CAG* coronary angiogram; *CRT* cardiac resynchronisation therapy

Unfortunately, not all areas of pre-procedural imaging can be covered by CTA and we need additional imaging techniques (as summarised in Fig. 1). For CRT patient selection, we need to assess left ventricular ejection fraction by echocardiogram or magnetic resonance imaging (MRI). These techniques have the advantage that they can also be used to identify myocardial scar and the area of latest mechanical activation. It has been shown in randomised trials that placing the left ventricular lead in the area of latest mechanical activation (out of scar) improves response rates to CRT and reduces hard clinical end points [5, 6]. In a perfect world, imaging would incorporate both venous anatomy and the ‘sweet spot’ for implantation showing the optimal target vein for implantation. Current studies are ongoing incorporating MRI information into intra-procedural fluoroscopy to guide implantation to the optimal myocardial area [7].

What should be done if pre-implantation imaging shows an unfavourable anatomy? Nguyên et al. have abandoned CRT implantation due to absence of a suitable target vein and changed the implantation strategy to a right-sided implant in two patients with persistent left-sided superior vena cava. If there is a class I indication for CRT, there are several alternative options for implantation. Left ventricular endocardial implantation is technically feasible and effective but carries a high risk of systemic thromboembolism even in patients using anticoagulation [8]. A safe alternative is the more invasive technique of epicardial left ventricular lead placement that in the hands of experienced thoracic surgeons can be done successfully with video-assisted thoracoscopic surgery [9].

Imaging does not stop at CRT implantation as shown in Fig. 1. Echocardiography should be used to determine the amount of reverse ventricular remodelling. It has been shown that the amount of reverse remodelling can be predicted by the relatively simple MARC score which incorporates indices of electrical and mechanical dyssynchrony [10]. Reverse remodelling predicts clinical outcome. In patients with suboptimal CRT response, additional measures might be taken to assess possibilities for improvement [11]. Even though atrioventricular interval optimisation has not been shown to improve CRT response, it could be effective in sub-optimal responders. Atrial remodelling has been shown to affect clinical outcome and is a possible target of atrioventricular interval optimisation [12]. It should also be investigated if the left ventricular lead has actually been placed in or near the area of latest mechanical activation. When using a quadripolar lead, the activation of multi-site pacing could be considered to improve reverse remodelling.

Pre-implantation as well as post-implantation cardiac imaging is crucial to optimise the response to CRT. We will see accumulating data that incorporate multi-modality cardiac imaging into implantation protocols to identify the optimal left ventricular pacing site. Implanting might become easier if good imaging techniques have been employed but CRT pathways in implanting centres need to incorporate alternative implantation strategies in patients with unfavourable venous anatomy.
